# Antibacterial and antibiofilm activities of pyocins produced by *Pseudomonas aeruginosa* against gram-negative bacteria: a systematic review (2015–2025)

**DOI:** 10.3389/fmicb.2026.1878395

**Published:** 2026-08-26

**Authors:** Sekari Hickson, Atinga Atimi, Adam Mustapha, Abubakar Shettima, Abdulrakib Abdulrahim, Ukpai Eze

**Affiliations:** 1 Department of Microbiology, Faculty of Life Sciences, University of Maiduguri, Maiduguri, Borno State, Nigeria; 2 Department of Public Health, Faculty of Health Sciences, Taraba State College of Health Technology, Takum, Nigeria; 3 Department of Medical Microbiology, Faculty of Medicine and Health Sciences, Universiti Putra Malaysia, Serdang, Selangor, Malaysia; 4 Chester Medical School, Faculty of Health, Medicine and Society, University of Chester, CH21BR, Chester, United Kingdom

**Keywords:** antimicrobial resistance, bacteriocin, biofilm, *Pseudomonas aeruginosa*, pyocin, systematic review

## Abstract

**Background:**

Antimicrobial resistance among Gram-negative bacteria, particularly *Pseudomonas aeruginosa*, *Escherichia coli*, *Klebsiella pneumoniae*, and *Acinetobacter baumannii*, has reduced the effectiveness of conventional antibiotics. Biofilm formation further increases bacterial tolerance and exacerbates resistance to multiple antibiotics. Pyocins, bacteriocins produced by *P. aeruginosa*, are emerging as promising targeted antibacterial and antibiofilm agents. However, there is no comprehensive systematic literature synthesis of pyocin extraction, characterization, antibacterial, and antibiofilm efficacy against a wide range of Gram-negative biofilm-forming pathogens.

**Objectives:**

To systematically evaluate evidence on the extraction, purification, characterization, antibacterial activity, antibiofilm efficacy, and resistance mechanisms of pyocins against Gram-negative bacteria.

**Methods:**

This review followed PRISMA 2020 guidelines and was registered in PROSPERO (ID: CRD420251064333). PubMed, Scopus, Web of Science, and CINAHL databases were searched for studies published between January 2015 and June 2025. Eligible studies included *in vitro* and preclinical investigations of S-, R-, F-type, or recombinant pyocins. Data on antibacterial activity and antibiofilm outcomes were extracted. Risk of bias was assessed using an adapted ROBINS-E framework. Findings were synthesized narratively.

**Results:**

A total of 191 records were identified; 137 were screened after duplicate removal, and 7 studies met the inclusion criteria. Pyocins demonstrated strong antibacterial activity against *P. aeruginosa*, with minimum inhibitory concentration values as low as 0.003 μg/mL. Antibiofilm effects ranged from 48 to 84% reduction, with enhanced activity when combined with enzymes, particularly glycoside hydrolases. Mechanisms of action included membrane depolarization, pore formation, and receptor-mediated uptake. Resistance mechanisms involved lipopolysaccharide modification, receptor mutation, and immunity proteins. Overall risk of bias was low to moderate.

**Conclusion:**

Pyocins exhibit potent antibacterial and antibiofilm activity and represent promising alternatives to conventional antibiotics. However, standardized methodologies, *in vivo* validation, and clinical studies are needed to support their therapeutic application.

## 1 Introduction

Antimicrobial resistance (AMR) is one of the most significant global public health challenges of the twenty-first century, with far-reaching consequences for both current and future healthcare systems (Gulumbe et al., 2023; Murray et al., 2022). Among resistant microorganisms, Gram-negative bacteria are particularly associated with high levels of multidrug resistance and limited therapeutic options (Murray et al., 2022; World Health Organization, 2024; Lu et al., 2012). Critical priority pathogens such as carbapenem-resistant *Pseudomonas aeruginosa* and *Acinetobacter baumannii*, alongside *Escherichia coli* and *Klebsiella pneumoniae*, are major causes of hospital-associated infections, including bloodstream infections, pneumonia, and urinary tract infections (World Health Organization, 2024; Cassini et al., 2019; Friedman et al., 2016). Resistance in these organisms is driven by intrinsic mechanisms such as efflux pumps and porin loss, as well as acquired determinants including *β*-lactamases and carbapenemases, reducing the effectiveness of conventional antibiotics (Murray et al., 2022; Blair et al., 2015; Olea-Ozuna et al., 2025).

Biofilm formation further exacerbates treatment failure by protecting bacteria within a self-produced extracellular polymeric substance (EPS), which limits antibiotic penetration and immune clearance. Biofilm-associated cells can tolerate antimicrobial concentrations far higher than planktonic cells, contributing to persistent infections in wounds, indwelling devices, and chronic respiratory diseases, particularly those involving *P. aeruginosa* and *A. baumannii* (Ciofu et al., 2022; Flemming and Wingender, 2010; Hall-Stoodley et al., 2004; Lebeaux et al., 2014; Mulcahy et al., 2014). This highlights the urgent need for alternative antimicrobial strategies. Bacteriocins, particularly pyocins produced by *P. aeruginosa*, have gained attention as potential alternatives to conventional antibiotics. Pyocins are classified into S-, R-, and F-types, with distinct mechanisms including nucleic acid degradation and membrane depolarization (Alqahtani et al., 2021; Ghequire and De Mot, 2014; Khalaf and Hussein, 2025; Michel-Briand and Baysse, 2002). Their high specificity and low impact on commensal microbiota make them promising candidates against multidrug-resistant pathogens (Scholl, 2017; Williams et al., 2008).

Methods for pyocin production typically involve mitomycin C induction followed by precipitation and chromatographic purification, although recombinant and heterologous expression systems are increasingly used to improve yield and scalability (Ghequire and De Mot, 2014; Grinter et al., 2012). However, variability in production and characterization methods limits comparability across studies. Recent studies also suggest that pyocins exhibit antibiofilm activity, particularly R-type pyocins, which can rapidly kill biofilm-associated cells through membrane disruption (Alqahtani et al., 2021; McCaughey et al., 2016; Oluyombo et al., 2019; Redero et al., 2018). However, most available evidence is based on planktonic or simplified biofilm models, and the lack of standardized antibiofilm assays limits direct comparison of findings. Despite increasing interest, there is no comprehensive synthesis of pyocin extraction, characterization, antibacterial and antibiofilm efficacy against Gram-negative biofilm-forming pathogens. Therefore, this systematic review evaluated methods for pyocin production and characterization; assessed their antibacterial and antibiofilm activity against Gram-negative biofilm-forming bacteria from 2015 to 2025, and highlighted knowledge gaps to inform future research and the potential clinical application of pyocins as novel antimicrobial agents for managing persistent biofilm-related infections.

## 2 Methods

### 2.1 Study protocol registration and reporting

This systematic review was conducted in accordance with the Preferred Reporting Items for Systematic Reviews and Meta-Analyses (PRISMA) 2020 guidelines (Page et al., 2021), and the review protocol was registered with the International Prospective Register of Systematic Reviews (PROSPERO) on 4 March 2026 under registration number CRD420251064333. The review focused on laboratory and preclinical studies evaluating the extraction, purification, characterization, antibacterial activity, and antibiofilm efficacy of pyocins derived from *P. aeruginosa* against biofilm-producing Gram-negative bacteria. The PRISMA checklist is provided in Supplementary file S1.

### 2.2 Review questions

The following research questions guided this systematic review:

1 What extraction and purification methods have been used to isolate pyocins from *P. aeruginosa*?
2 What structural and biochemical characterization techniques have been employed to define pyocin types (S-, R-, and F-type)?
3 What is the antibacterial efficacy of pyocins against Gram-negative bacterial pathogens?
4 What evidence exists regarding the antibiofilm activity of pyocins?
5 What resistance mechanisms, if any, have been reported against pyocins?

### 2.3 Review framework (PICO)

Table 1 outlines the PICO framework (population, exposure, comparator/context, outcome) guiding this review.

**Table 1:** The PICO framework (Population, Exposure, Comparator/Context, Outcome) guiding this review.

Component	Description
Population (P)	Gram-negative bacteria, including clinical and laboratory strains such as *P. aeruginosa*, *E. coli*, *K. pneumoniae*, and *A. baumannii*.
Intervention (I)	Pyocins (S-type, R-type, F-type) extracted or recombinantly produced from *P. aeruginosa*.
Comparator (C)	Conventional antibiotics, untreated controls, or other bacteriocins, where applicable.
Outcome (O)	Antibacterial activity (e.g., MIC, MBC, zone of inhibition), antibiofilm activity (e.g., MBEC, biofilm biomass reduction, EPS reduction), structural characterization, and resistance mechanisms.

MIC, minimum inhibitory concentration; MBC, minimum bactericidal concentration; MBEC, minimum biofilm eradication concentration; EPS, extracellular polymeric substances.

### 2.4 Eligibility criteria

Studies were screened according to the criteria outlined in the Table 2 using the Rayyan online systematic review management tool. Further selection was based on study design, with all included studies categorized by the experimental methodologies used to evaluate pyocin extraction, characterization, and antibacterial and antibiofilm activities. Final inclusion was determined based on studies that assessed the effects of pyocins on Gram-negative bacteria using standardized laboratory or preclinical approaches.

**Table 2:** Inclusion and exclusion criteria.

Criterion	Inclusion criteria	Exclusion criteria
Study design / type	Experimental, laboratory-based, or preclinical studies	Review articles, systematic reviews, meta-analyses, editorials, letters, conference abstracts, commentaries, and study protocols
Publication period	January 1, 2015–June 30, 2025	Studies published outside the specified period
Intervention focus	Studies reporting the extraction, purification, or recombinant production of pyocins from *P. aeruginosa*	Studies not involving pyocins (e.g., other bacteriocins without pyocin evaluation)
Antibacterial assessment	Studies evaluating antibacterial activity against Gram-negative bacteria	Studies not reporting antibacterial activity
Antibiofilm assessment	Studies assessing antibiofilm activity using standardized methods (e.g., microtiter biofilm assays, crystal violet staining, MBEC, confocal microscopy)	Studies not reporting antibiofilm activity
Outcome measures	Quantitative outcomes (e.g., MIC values, log reduction, % biofilm inhibition)	Studies lacking quantitative antibacterial or antibiofilm outcomes
Scope of study	Functional studies assessing antibacterial and/or antibiofilm efficacy	Studies focused solely on genetic regulation without functional antibacterial testing
Publication type	Peer-reviewed original research articles	Non-peer-reviewed publications
Language	No language restriction	-

### 2.5 Search strategy

A comprehensive literature search was conducted in PubMed, Scopus, Web of Science, and CINAHL databases for peer-reviewed articles published from January 2015 to June 2025. The search strategy combined keywords and database-specific subject headings related to pyocins, *P. aeruginosa*, biofilms, Gram-negative bacteria, and antibacterial or antibiofilm activity. A representative Scopus search syntax was: (pyocin OR “S-type pyocin” OR “R-type pyocin”) AND (“*Pseudomonas aeruginosa*” OR “*P. aeruginosa*”) AND (biofilm* OR sessile OR “surface-attached”) AND (“Gram-negative bacteria” OR “*Escherichia coli*” OR “*Klebsiella pneumoniae*” OR “*Acinetobacter baumannii*”) AND (antibiofilm* OR antibacteri* OR antimicrobial*). The complete search strategies for all databases are provided in Supplementary file S2.

### 2.6 Study selection

**Figure 1:**
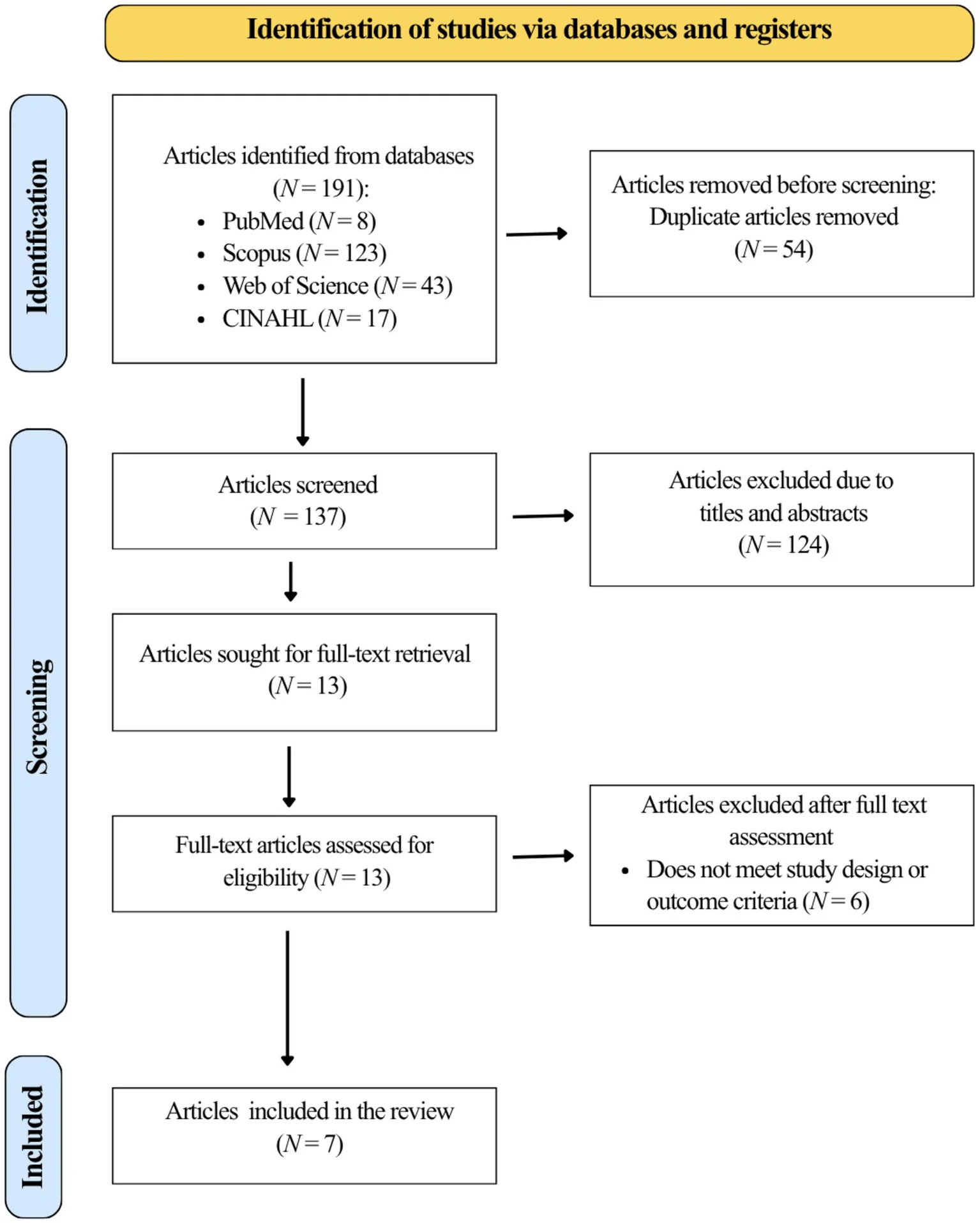
PRISMA flow diagram for the systematic review and meta-analysis showing the identification, screening, eligibility, and inclusion of studies.

All retrieved records were imported into the Rayyan online systematic review management tool for screening and automatic removal of duplicates. Two reviewers independently screened titles and abstracts for relevance based on the predefined eligibility criteria. Full-text screening was conducted independently by two additional reviewers. Disagreements were resolved through discussion and consensus, with arbitration by a third reviewer when necessary. The study selection process is illustrated in the PRISMA flow diagram (Figure 1).

### 2.7 Risk-of-bias assessment

In the absence of standardized tools for *in vitro* studies, the risk of bias (internal validity) was assessed using an adapted framework informed by existing guidance (Tran et al., 2021). An adapted version of the ROBINS-E tool (Higgins et al., 2024) was used for experimental laboratory studies (Sterne et al., 2016). The assessment considered key domains, including study experimental design, sample size adequacy, methodological rigor, outcome measurement, and reporting transparency. Studies were classified as having low, moderate, or high risk of bias based on overall performance across these domains (See Supplementary file S3).

### 2.8 Data extraction

Data were extracted using a piloted, standardized data-extraction form to ensure consistency and reproducibility across studies (Supplementary file S4). For each included study, information was collected on the author(s) and year of publication, country and laboratory setting, study design, and experimental model. Details on the pyocin type (S-, R-, or F-type), extraction and purification methods, and characterization techniques (e.g., SDS-PAGE, electron microscopy, mass spectrometry) were recorded. Data on target bacterial strains, antibacterial outcomes (including minimum inhibitory concentration [MIC], minimum bactericidal concentration [MBC], zone of inhibition, and log reduction), and antibiofilm outcomes (minimum biofilm eradication concentration [MBEC] and percentage biofilm reduction) were also extracted. Any reported resistance mechanisms or receptor specificities were noted. When multiple outcome measures were reported, the most comprehensive dataset was selected. Data extraction was performed independently by two reviewers, with discrepancies resolved through discussion and consensus. The final dataset was used for narrative synthesis and, where appropriate, quantitative summarization of the findings.

### 2.9 Data synthesis

Data from the included studies were synthesized using a structured narrative approach. Extraction and purification techniques were summarized to highlight methodological variations across studies, including chromatography, precipitation, and ultrafiltration. Antibacterial and antibiofilm outcomes were tabulated to allow direct comparison of minimum inhibitory concentrations (MICs), minimum bactericidal concentrations (MBCs), zone of inhibition, log reductions, and biofilm eradication metrics (MBEC, percentage biofilm reduction). Comparisons between pyocin types (S-, R-, and F-type) were made to evaluate differences in spectrum of activity and potency. Additionally, reported resistance mechanisms, receptor specificity, and patterns of reduced susceptibility were identified and discussed to contextualize pyocin efficacy and potential limitations in targeting Gram-negative biofilm-producing bacteria. The final synthesis integrated these elements to provide a comprehensive overview of current laboratory and preclinical evidence.

## 3 Results

### 3.1 Study selection

The systematic search identified 191 records from four electronic databases: CINAHL (*N* = 17), Web of Science (*N* = 43), PubMed (*N* = 8), and Scopus (*N* = 123). After removing 54 duplicate records, 137 unique articles remained and underwent primary screening based on titles and abstracts. During the title and abstract screening stage, 124 articles were excluded for not meeting the predefined eligibility criteria. Subsequently, 13 articles were sought for full-text retrieval. Six articles were removed after full-text assessment for not meeting the study design or outcome criteria, and seven were included in the final review (Alqahtani et al., 2021; Khalaf and Hussein, 2025; McCaughey et al., 2016; Oluyombo et al., 2019; Mei et al., 2021; Behrens et al., 2020; Dingemans et al., 2016). The detailed study selection process, including identification, screening, eligibility assessment, and final inclusion, is presented in the PRISMA 2020 flow diagram (Figure 1).

### 3.2 Study characteristics

The seven articles collected in the review exhibit a fairly high level of geographical representation, experimental study designs, and sample sources, but the focus is on *P. aeruginosa.* Most of the literature was published in high-income nations, especially the United States and the United Kingdom, with additional studies in Iraq and Belgium, indicating international and regional interest and capacity in pyocin-based therapies. Each study employed an experimental design (primarily *in vitro*), with studies, including *in vivo* murine models, to assess the efficacy of the therapy (Alqahtani et al., 2021; McCaughey et al., 2016). This combination of laboratory and animal models enhances the translational applicability of the results, especially for wound and lung infections. The sample sizes were highly heterogeneous. Some studies examined comparatively small numbers of clinical isolates (e.g., 2063 isolates), whereas others examined large-scale genomic datasets, e.g., the study by Mei *et al*. examined more than 800 strains in silico (Table 3; Mei et al., 2021). Clinical isolates were mainly obtained from cystic fibrosis (CF) patients and burn or wound infections, both of which are closely linked to chronic biofilm formation by *P. aeruginosa*. It is worth noting that isolates were specifically selected by Oluyombo et al. for biofilm-forming capability (Oluyombo et al., 2019), whereas other researchers did not directly measure biofilm production and instead assessed antimicrobial susceptibility or genetic characteristics.

**Table 3:** Study characteristics.

Author(s)	Country	Study design	Sample size	Source of isolates	Bacterial species	Biofilm-producing strains	Study setting
Alqahtani et al. (2021)	USA	Experimental (*In vitro & In vivo*)	52 clinical isolates + lab strains + murine model	CF (23), burn (26), lab (3) = 52 isolates	*P. aeruginosa*	5 sensitive strains	Lab + Animal
Mei et al. (2021)	USA	Experimental (*In vitro*)	852 in silico + 183 isolates	IPCD + CF patients	*P. aeruginosa*	Not specified	Lab
Oluyombo et al. (2019)	UK	Experimental (*In vitro*)	24 CF isolates	CF patients	*P. aeruginosa*	24 (biofilm forming)	Lab
Khalaf and Hussein (2025)	Iraq	Experimental (*In vitro*)	20 isolates	Wounds & burns	*P. aeruginosa*	6 strong biofilm producers	Lab
McCaughey et al. (2016)	UK	Experimental (*In vitro & In vivo*)	Isolates + murine model	Clinical & environmental	*P. aeruginosa*	Not specified	Lab + Animal
Behrens et al. (2020)	UK	Experimental (*In vitro*)	Not specified	Lab strains + *E. coli*	*P. aeruginosa*, *E. coli*	Not specified	Lab
Dingemans et al. (2016)	Belgium	Experimental (*In vitro*)	110 CF isolates	CF patients	*P. aeruginosa*	Not specified	Lab

### 3.3 Narrative synthesis regarding pyocin methods

The methods used for pyocin production, extraction, purification, and characterization in the included studies varied significantly (Table 4) depending on the type of pyocin, the experimental aim, and the method used. Two major types of pyocins were examined, namely, R-type pyocins (phage tail-like bacteriocins) and S-type pyocins (colicin-like protein bacteriocins). R-type pyocins were investigated more often in the framework of biofilm inhibition and inter-strain competition (Alqahtani et al., 2021; Oluyombo et al., 2019). S-type pyocins were more commonly related to molecular, functional, and structural characterization, such as receptor binding and translocation processes (McCaughey et al., 2016; Behrens et al., 2020). Moreover, there are studies on genotypic characterization that have not been derived from experiments, but emphasize the diversity of pyocin distribution (Mei et al., 2021). The induction techniques varied depending on whether native or recombinant systems were used. Mitomycin C was commonly used to cause pyocin production of *P. aeruginosa*, which is in line with the effect of this medication in activating bacterial SOS response (Khalaf and Hussein, 2025; Oluyombo et al., 2019). Conversely, those recombinant expression systems employed in *E.coli* used inducible promoters, such as Isopropyl *β*-D-1-thiogalactopyranoside (IPTG) or arabinose, to overexpress specific pyocin subtypes (Alqahtani et al., 2021; McCaughey et al., 2016; Behrens et al., 2020; Dingemans et al., 2016). Regarding the extraction methods, the results showed relative similarity, as the crude lysate was usually prepared by centrifugation, followed by chloroform treatment (Alqahtani et al., 2021; Khalaf and Hussein, 2025; Oluyombo et al., 2019). Nevertheless, there was a significant difference in purification strategies. Some of the studies used more refined techniques like ammonium C precipitation and ultracentrifugation (Alqahtani et al., 2021; Oluyombo et al., 2019), whereas others utilized crude pyocin extracts (Khalaf and Hussein, 2025). Recombinant S-type pyocins were normally purified by affinity chromatography and size-exclusion chromatography, which is indicative of the increased purity required in structural and mechanistic studies (McCaughey et al., 2016; Behrens et al., 2020; Dingemans et al., 2016). The methods of characterization included simple microbiological tests, such as zone-of-inhibition and spot tests (Alqahtani et al., 2021; Khalaf and Hussein, 2025; Oluyombo et al., 2019), as well as complex molecular and biophysical methods. These were polymerase chain reaction (PCR), whole-genome sequencing and genotyping using bioinformatics analyses (Mei et al., 2021; Dingemans et al., 2016), and X-ray crystallography, isothermal titration calorimetry (ITC), small-angle X-ray scattering (SAXS), surface plasmon resonance (SPR), and circular dichroism (CD) spectroscopy to study the structure and functionality (McCaughey et al., 2016).

**Table 4:** Pyocin production, extraction, and characterization methods.

Author(s)	Pyocin type	Induction method	Extraction method	Purification method	Characterization techniques
Alqahtani et al. (2021)	R2 (recombinant)	0.01% arabinose in *E. coli* BL21	Differential centrifugation; chloroform	Ammonium sulfate precipitation; ultracentrifugation; heat treatment	SDS-PAGE, Bradford assay, inhibition assay
Mei et al. (2021)	R1, R2, R5 (genotyping only)	Not applicable	Not applicable	Not applicable	BLAST, multiplex PCR, whole-genome sequencing
Oluyombo et al. (2019)	R1, R2	Mitomycin C (3 μg/mL)	Centrifugation; chloroform	Ammonium sulfate precipitation; ultracentrifugation	Spot test, PCR, mutagenesis
Khalaf and Hussein (2025)	Unspecified pyocin	Mitomycin C (2 μg/mL)	Centrifugation; chloroform	None (crude extract)	Cup assay, well diffusion, Lowry protein assay
McCaughey et al. (2014)	S-type (SD1–SD3)	1.0 mM IPTG	Sonication	Ni-affinity chromatography; gel filtration	ITC, SAXS, CD spectroscopy, mutagenesis
Behrens et al. (2020)	S5 (pore-forming)	IPTG induction	Sonication	Ni-affinity chromatography; size exclusion chromatography	X-ray crystallography, SPR, SAXS, fluorescence microscopy
Dingemans et al. (2016)	S6 (rRNase)	IPTG induction	French press	Ni-affinity chromatography	SDS-PAGE, MIC, PCR, RT-PCR, modeling

IPTG, isopropyl β-D-1-thiogalactopyranoside; SDS-PAGE, sodium dodecyl sulfate–polyacrylamide gel electrophoresis; BLAST, basic local alignment search tool; multiplex PCR, multiplex polymerase chain reaction; WGS, whole genome sequencing; PCR, polymerase chain reaction; ITC, isothermal titration calorimetry; SAXS, small-angle X-ray scattering; CD spectroscopy, circular dichroism spectroscopy; SPR, surface plasmon resonance; MIC, minimum inhibitory concentration; RT-PCR, reverse transcription polymerase chain reaction.

### 3.4 Narrative synthesis of key findings

The results of the included studies are consistent with the idea that pyocins have a strong antibacterial effect against *P. aeruginosa*, but their effectiveness varies with strain sensitivity, pyocin type, and environmental factors (Table 5). The R-type pyocins exhibited high bactericidal activity, with some studies reporting very low MICs. For example, Alqahtani et al. have reported high activity against both lab- and clinic-isolated strains, though a percentage of strains have shown partial or complete resistance (Scholl, 2017). Similarly, Oluyombo et al. demonstrated that R-type pyocins are essential for intra-species competition, especially in biofilm habitats, where they modulate strain dominance (McCaughey et al., 2016). Some studies focused mainly on antibiofilm activity (Alqahtani et al., 2021; Khalaf and Hussein, 2025; Oluyombo et al., 2019). Pyocins have been shown to prevent biofilm growth and destroy pre-existing biofilms (Alqahtani et al., 2021; Khalaf and Hussein, 2025; Oluyombo et al., 2019). Specifically, Alqahtani et al. showed that recombinant R2 pyocin preparations removed early-stage biofilms, with better results obtained when combined with glycoside hydrolases. On the same note, Khalaf and Hussein reported a biofilm inhibition range of 48–67.7 percent with crude pyocin extracts (Khalaf and Hussein, 2025). Nevertheless, there was greater resistance to pyocin treatment in mature biofilms, and it is essential to highlight that biofilms should be treated with a combination approach to achieve successful outcomes. The pyocins of the S-type exhibited varying and highly specific modes of action, specifically the pore formation, ribonuclease activity and receptor-mediated uptake. Indicatively, Behrens et al. defined pyocin S5 as a pore-forming toxin, which needs to bind to lipopolysaccharide (LPS) components and receptors on the outer membrane (Behrens et al., 2020). Similarly, Dingemans et al. (2016) found that pyocin S6 is a 16S rRNA-degrading enzyme that can inhibit vulnerable strains by selectively degrading 16S RNA. Moreover, McCaughey et al. have shown that S-type pyocins are mediated by TonB-dependent transport systems and specific receptors (e.g., FpvAI), indicating their specificity of targeting (McCaughey et al., 2016). Structural and mechanistic investigations also indicated that pyocins use a multi-step importation process, with an initial step of binding to lipopolysaccharides (LPS) and translocation via outer membrane transporters (McCaughey et al., 2016; Behrens et al., 2020). The results highlight the specificity and efficiency of bacterial killing through pyocins. Pyocin resistance was also demonstrated in various studies and explained by changes in LPS structure, loss or mutation of outer membrane receptors, and the presence of immunity genes, which help confer resistance to self-killing (Khalaf and Hussein, 2025; Oluyombo et al., 2019; Mei et al., 2021; Dingemans et al., 2016). Interestingly, Mei et al. identified heterogeneous susceptibility within the clonal population, indicating that microevolutionary adaptation, particularly to LPS phenotypes, can substantially impact pyocin susceptibility in clinical isolates (Mei et al., 2021).

**Table 5:** Antibacterial and antibiofilm activity of pyocins.

Author(s)	Target organism	Antibacterial activity	Antibiofilm activity	Resistance observed	Key outcome
Alqahtani et al. (2021)	*P. aeruginosa* (clinical and lab strains)	Highly potent (MIC 0.003 μg/mL); 31% sensitive	Inhibits and eradicates biofilms; enhanced with enzymes	31% resistant; mature biofilms are resistant alone	Promising topical therapy
Mei et al. (2021)	*P. aeruginosa* (CF isolates)	Variable susceptibility	Not tested	Lipopolysaccharide structural variation	Heterogeneous response
Oluyombo et al. (2019)	*P. aeruginosa* (CF isolates)	Strong antimicrobial effect	Eradicates established biofilms	Immunity genes confer resistance	Biofilm competition role
Khalaf and Hussein (2025)	*P. aeruginosa* (clinical isolates)	Moderate antibacterial activity	48–67.7% biofilm inhibition	Self-immunity	Potential alternative therapy
McCaughey et al. (2016)	*P. aeruginosa*	Highly potent	Not tested	Receptor mutation	Novel killing mechanism
Behrens et al. (2020)	*P. aeruginosa*, *E. coli*	Potent pore-forming activity	Not tested	Common polysaccharide antigen and receptor dependency	Mechanism of import identified
Dingemans et al. (2016)	*P. aeruginosa* (CF isolates)	Moderate activity (~20% susceptible)	Not tested	Immunity genes (pys6)	Novel S6 pyocin identified

CF, cystic fibrosis; MIC, minimum inhibitory concentration.

### 3.5 Risk of bias narrative summary

The maximum risk of bias across the selected studies was low to moderate, and the majority of the studies were methodologically sound and had valid outcome measures (Supplementary Table S4). Most of the studies employed well-designed experimental designs, particularly those that used both *in vitro* and *in vivo* approaches, thereby making their results more valid and reproducible. The risk of bias was identified as low in such works as Alqahtani et al. and McCaughey et al. due to the extensive experimental designs, which included several validation procedures, strong outcome measures, and the incorporation of *in vivo* models (Alqahtani et al., 2021; McCaughey et al., 2016). Oluyombo et al. demonstrated high methodological rigor through well-constructed biofilm and competition assays, which contributed to a low total bias rating (Oluyombo et al., 2019). The report by Behrens et al. and Dingemans et al. also had a low risk of bias because they employed extensive analytical methods and a comprehensive characterization of functional aspects (Behrens et al., 2020; Dingemans et al., 2016). Mei et al. and Khalaf and Hussein, on the contrary, were evaluated as having a moderate risk of bias (Khalaf and Hussein, 2025; Mei et al., 2021). In the case of Mei et al., it was in silico genomic analysis and a lack of functional biofilm assays that restricted the scope of outcome analysis. Likewise, Khalaf and Hussein employed rather simplistic methodologies, such as crude pyocin extracts without a significant purification step, and a small sample size, which may affect the overall generalizability and accuracy of the results (Khalaf and Hussein, 2025). There were also differences in the adequacy of the sample size in the research. Although other studies involved a large amount of data or several isolates (e.g., Mei et al., Dingemans et al.), other studies used smaller sample sizes or no sample size at all, which risks selection bias (Mei et al., 2021; Dingemans et al., 2016). Also, disparity in outcome measurement methods, especially the lack of standardized biofilm tests in certain studies, also contributed toward heterogeneity in the quality of evidence.

## 4 Discussion

### 4.1 Overview of pyocin activity and antimicrobial potential

This systematic review synthesized existing information on the production, characterization, and antimicrobial activity of pyocins produced by *P. aeuroginosa* against a wide range of Gram-negative bacterial pathogens and integrates it with the broader literature on bacteriocins and precision antimicrobials. Overall, the included studies indicate therapeutic value, as pyocins are very potent, strain-specific antibacterial agents with potential uses in chronic and biofilm-associated infections. The antimicrobial efficacy of pyocins is high in the studies considered here, and this is in line with previous preparatory literature on bacteriocins, which has shown that phage tail-like bacteriocins are capable of causing rapid membrane depolarization and cell mortality (Ghequire and De Mot, 2014; Michel-Briand and Baysse, 2002). R-type pyocins, specifically, have been shown to exert bactericidal effects at very low concentrations, which are usually comparable to or even higher than those of traditional antibiotics (Williams et al., 2008; Scholl and Martin, 2008). This strengthens their potential as precision antimicrobials, particularly against the backdrop of increasing AMR, where targeted therapies are becoming a more popular choice than broad-spectrum agents (Abedon, 2018).

### 4.2 Antibiofilm activity and therapeutic relevance

One of the key findings of this review is that pyocins exhibit high antibiofilm activity. This is in line with past research indicating that bacteriocins can enter and destroy biofilm structures, which are usually not susceptible to antibiotic therapy (Hall-Stoodley et al., 2004). Biofilm-related infections, especially those in CF and chronic wounds, represent a significant clinical problem in *P. aeruginosa* infections because of the protective EPS matrix (Bjarnsholt, 2013). In line with earlier reports, the incorporated articles demonstrate that pyocins are more effective against early-stage biofilms compared to mature ones, likely due to diffusion limitations and metabolic heterogeneity in established biofilms (Stewart and Franklin, 2008). The increased pyocin activity in the presence of biofilm-degrading enzymes (e.g., aureolysin, proteinase K, V8 serine protease) supports the literature advocating combination therapies as a more effective approach to biofilm elimination (Fleming and Rumbaugh, 2017).

### 4.3 Mechanisms of action, resistance, and methodological advances

Mechanistically, the evidence in this review supports previously established models of bacteriocin action (Behrens et al., 2017). It was demonstrated that S-type pyocins employ receptor-mediated uptake systems such as TonB-dependent transporters, after initial interaction with lipopolysaccharides (LPS). This “Trojan horse” mechanism has been widely described in colicins and related bacteriocins (Ghequire and De Mot, 2014; Behrens et al., 2017). Structural analyses also show highly specific interactions with outer membrane receptors, followed by translocation into the periplasm or cytoplasm (McCaughey et al., 2016; Behrens et al., 2020). This specificity underpins pyocin killing efficiency but also contributes to strain-dependent susceptibility.

The development of resistance to pyocins observed in some included studies is consistent with previous findings. Mechanisms of resistance include alterations in LPS structure, loss or mutation of outer membrane receptors, and immunity genes in producer strains (Ghequire and De Mot, 2014; Michel-Briand and Baysse, 2002). Similar resistance patterns have been reported in bacteriophage therapy, suggesting convergent evolutionary responses (Labrie et al., 2010). Notably, some studies indicate that resistance may carry a fitness cost, including reduced virulence or nutrient acquisition, which may be exploitable therapeutically (Ghequire and De Mot, 2014).

Methodological heterogeneity was also evident across studies. Earlier work relied on crude extraction and basic microbiological assays, whereas recent studies have employed advanced techniques such as whole-genome sequencing, X-ray crystallography, and biophysical analyses, enabling improved structure–function characterization (Behrens et al., 2020; Cascales et al., 2007). This shift has facilitated the development of engineered pyocins with enhanced activity and altered specificity, which is an emerging research direction (Scholl, 2017).

### 4.4 Translational relevance, global context, and limitations

The translational relevance of pyocins is supported by *in vivo* studies showing reduced bacterial burden and improved survival in infection models, particularly pulmonary and wound infections (Williams et al., 2008). However, a significant gap in clinical trials remains, limiting direct clinical application (Abedon, 2018).

On a global scale, most studies originate from high-income countries, highlighting unequal research capacity despite the substantial burden of Gram-negative bacterial infections in low- and middle-income regions (World Health Organization, 2024; Alqahtani et al., 2021; McCaughey et al., 2016; Oluyombo et al., 2019; Mei et al., 2021; Dingemans et al., 2016). Previous reports emphasize the need for context-specific research and capacity building to address antimicrobial resistance globally (World Health Organization, 2023). Although no language restrictions were applied during the search process, the databases used primarily index English-language literature, and no non-English databases were searched. Therefore, relevant studies published in other languages or indexed in regional databases may not have been captured.

Notably, none of the included studies investigated F-type pyocins. Their underrepresentation likely reflects a combination of structural complexity, extensive genetic diversity, low production yields, limited availability of recombinant expression systems, delayed mechanistic characterization, and a historical research emphasis on the more extensively studied R- and S-type pyocins. In addition, the uneven distribution of F-type pyocin genes among *P. aeruginosa* isolates and variability in LPS receptors that determine susceptibility have further hindered systematic investigation. Nevertheless, recent structural and functional studies are beginning to address these limitations, demonstrating that F-type pyocins are structurally distinct from R-type pyocins and exhibit bactericidal activity comparable to that of R-type pyocins (Saha et al., 2023). This represents an important knowledge gap that warrants further investigation to fully define their therapeutic potential. Other limitations should be considered. The heterogeneity of study designs, variability in sample sizes, and lack of standardized biofilm assays limited comparability across studies. In addition, only a small number of eligible studies were identified over the 10-year search period, likely reflecting the limited translation of pyocin research into therapeutic applications, the technical challenges associated with pyocin extraction, purification, and characterization, and the scarcity of *in vivo* and clinical investigations. Consequently, the evidence base remains sparse and heterogeneous, precluding meaningful subgroup analyses according to pyocin subtype, purification protocol, biofilm maturity stage, or experimental model. These limitations reduce the generalizability of the findings and constrain translational inference. Therefore, the therapeutic potential of pyocins should be interpreted cautiously until supported by additional standardized preclinical and clinical studies.

## 5 Conclusion

Pyocins represent a promising class of next-generation, targeted antimicrobials with potent antibacterial and antibiofilm activity against Gram-negative bacteria, especially *P. aeruginosa*. Their specificity, diverse mechanisms of action, and effectiveness against resistant strains position them as potential candidates for future therapeutic development. However, overcoming challenges related to resistance, delivery, and clinical translation will be essential for their successful integration into modern antimicrobial strategies.
