# Supplementary material for: Antibacterial and antibiofilm activities of pyocins produced by Pseudomonas aeruginosa against gram-negative bacteria: a systematic review (2015–2025)
Source: Front Microbiol. 2026 Aug 26;17:1878395. doi: 10.3389/fmicb.2026.1878395 (PMC13553936; doi:10.3389/fmicb.2026.1878395)
Supplement: Supplementary file 1 [file Data_Sheet_1.PDF]

SEARCH STRATEGY

Extraction and Characterization of Pyocins from Pseudomonas aeruginosa and Their Activity Against Biofilm-Forming Gram-Negative Bacteria: A Systematic Review (2015–2025)

(pyocin OR pyocins OR "S-type pyocin" OR "R-type pyocin" OR "F-type pyocin"

OR bacteriocin* OR "bacteriocin-like" OR "bacteriocin like" OR "bactericidal protein*" OR "bactericidal peptide*" OR colicin* OR microcin*)

AND

("Pseudomonas aeruginosa" OR "P. aeruginosa" OR pseudomonas)

AND

(biofilm* OR "bio-film" OR "bio film"

OR sessile OR "surface-attached" OR "surface attached"

OR "microbial biofilm*" OR "bacterial biofilm*")

AND

("Gram-negative" OR "Gram negative" OR "gram-negative bacteria" OR "gram negative bacteria" OR "GN bacteria" OR Enterobacteriaceae OR Proteobacteria OR "Escherichia coli" OR "Klebsiella pneumoniae" OR "Acinetobacter baumannii" OR "Salmonella spp" OR "Shigella spp")

AND

(extract* OR isolat* OR purif* OR purification OR characteriz* OR characterization OR "physicochemical characterization" OR "protein characterization" OR "structural characterization" OR antibiofilm* OR "anti-biofilm" OR antibacteri* OR antimicrobial* OR "minimum inhibitory concentration" OR MIC OR "minimum bactericidal concentration" OR MBC OR MBEC OR "minimum biofilm eradication concentration" OR "zone of inhibition" OR bactericidal OR bacteriostatic)

Pubmed search

S/N	**SEARCH**	**Number**
#1	(pyocin[Title/Abstract] OR pyocins[Title/Abstract] OR "S-type pyocin"[Title/Abstract] OR "R-type pyocin"[Title/Abstract] OR "F-type pyocin"[Title/Abstract] OR bacteriocin*[Title/Abstract] OR bacteriocidal protein*[Title/Abstract] OR bacteriocidal peptide*[Title/Abstract] OR bacteriocin-like[Title/Abstract]) Filters: in the last 10 years	3,572
#2	(Pseudomonas aeruginosa[Title/Abstract] OR P. aeruginosa[Title/Abstract] OR pseudomonas[Title/Abstract]) Filters: in the last 10 years	57,811
#3	(biofilm*[Title/Abstract] OR "bio-film"[Title/Abstract] OR "bio film"[Title/Abstract] OR sessile[Title/Abstract] OR "surface-attached"[Title/Abstract]) Filters: in the last 10 years	67,094
#4	("Gram-negative"[Title/Abstract] OR gram negative[Title/Abstract] OR GN bacteria[Title/Abstract] OR "Enterobacteriaceae"[Title/Abstract] OR "Proteobacteria"[Title/Abstract]) Filters: in the last 10 years	75,983
#5	(extract*[Title/Abstract] OR isolat*[Title/Abstract] OR purif*[Title/Abstract] OR purification[Title/Abstract] OR characteriz*[Title/Abstract] OR characterisation[Title/Abstract] OR "physicochemical"[Title/Abstract] OR "protein characterization"[Title/Abstract] OR "structural characterization"[Title/Abstract] OR "antibiofilm"[Title/Abstract] OR antibiofilm[Title/Abstract] OR antibio-film[Title/Abstract] OR antibiofilm*[Title/Abstract] OR antibacteri*[Title/Abstract] OR antimicrobial*[Title/Abstract] OR "minimum inhibitory concentration"[Title/Abstract] OR MIC[Title/Abstract] OR MBEC[Title/Abstract] OR "minimum biofilm eradication concentration"[Title/Abstract]) Filters: in the last 10 years	2,284,913

#6	Search: (((((pyocin[Title/Abstract] OR pyocins[Title/Abstract] OR "S-type pyocin"[Title/Abstract] OR "R-type pyocin"[Title/Abstract] OR "F-type pyocin"[Title/Abstract] OR bacteriocin*[Title/Abstract] OR bacteriocidal protein*[Title/Abstract] OR bacteriocidal peptide*[Title/Abstract] OR bacteriocin-like[Title/Abstract]) AND (y_10[Filter])) AND ((Pseudomonas aeruginosa[Title/Abstract] OR P. aeruginosa[Title/Abstract] OR pseudomonas[Title/Abstract]) AND (y_10[Filter]))) AND ((biofilm*[Title/Abstract] OR "bio-film"[Title/Abstract] OR "bio film"[Title/Abstract] OR sessile[Title/Abstract] OR "surface-attached"[Title/Abstract]) AND (y_10[Filter]))) AND (("Gram-negative"[Title/Abstract] OR gram negative[Title/Abstract] OR GN bacteria[Title/Abstract] OR "Enterobacteriaceae"[Title/Abstract] OR "Proteobacteria"[Title/Abstract]) AND (y_10[Filter]))) AND ((extract*[Title/Abstract] OR isolat*[Title/Abstract] OR purif*[Title/Abstract] OR purification[Title/Abstract] OR characteriz*[Title/Abstract] OR characterisation[Title/Abstract] OR "physicochemical"[Title/Abstract] OR "protein characterization"[Title/Abstract] OR "structural characterization"[Title/Abstract] OR "antibiofilm"[Title/Abstract] OR antibiofilm[Title/Abstract] OR antibio-film[Title/Abstract] OR antibiofilm*[Title/Abstract] OR antibacteri*[Title/Abstract] OR antimicrobial*[Title/Abstract] OR "minimum inhibitory concentration"[Title/Abstract] OR MIC[Title/Abstract] OR MBEC[Title/Abstract] OR "minimum biofilm eradication concentration"[Title/Abstract]) AND (y_10[Filter])) Filters: 2015 to 2025	8

Scopus search

S/N	**SEARCH**	**Number**
#1	TITLE-ABS-KEY (pyocin OR pyocins OR "S-type pyocin" OR "R-type pyocin" OR "F-type pyocin" OR bacteriocin* OR bacteriocidal protein* OR bacteriocidal peptide* OR bacteriocin-like)	3,003
#2	TITLE-ABS-KEY (Pseudomonas aeruginosa OR P. aeruginosa OR pseudomonas)	194,506
#3	TITLE-ABS-KEY (biofilm* OR "bio-film" OR "bio film" OR sessile OR "surface-attached")	179,673
#4	TITLE-ABS-KEY ("Gram-negative" OR gram negative OR GN bacteria OR "Enterobacteriaceae" OR "Proteobacteria")	180,422
#5	TITLE-ABS-KEY (extract* OR isolat* OR purif* OR purification OR characteriz* OR characterisation OR "physicochemical" OR "protein characterization" OR "structural characterization" OR "antibiofilm" OR antibiofilm OR antibio-film OR antibiofilm* OR antibacteri* OR antimicrobial* OR "minimum inhibitory concentration" OR MIC OR MBEC OR "minimum biofilm eradication concentration")	13,027,138
#6	(TITLE-ABS-KEY (pyocin OR pyocins OR "S-type pyocin" OR "R-type pyocin" OR "F-type pyocin" OR bacteriocin* OR bacteriocidal protein* OR bacteriocidal peptide* OR bacteriocin-like)) AND (TITLE-ABS-KEY (Pseudomonas aeruginosa OR P. aeruginosa OR pseudomonas)) AND (TITLE-ABS-KEY (biofilm* OR "bio-film" OR "bio film" OR sessile OR "surface-attached")) AND (TITLE-ABS-KEY ("Gram-negative" OR gram negative OR GN bacteria OR "Enterobacteriaceae" OR "Proteobacteria")) AND (TITLE-ABS-KEY (extract* OR isolat* OR purif* OR purification OR characteriz* OR characterisation OR "physicochemical" OR "protein characterization" OR "structural characterization" OR "antibiofilm" OR antibiofilm OR antibio-film OR antibiofilm* OR antibacteri* OR	123

	antimicrobial* OR "minimum inhibitory concentration" OR MIC OR MBEC OR "minimum biofilm eradication concentration")) AND PUBYEAR > 2014 AND PUBYEAR < 2026	

CINAHL

TX (pyocin OR pyocins OR “S-type pyocin” OR “R-type pyocin” OR “F-type pyocin” OR bacteriocin* OR “bacteriocin-like” OR colicin* OR microcin* OR “bactericidal protein*” OR “bactericidal peptide*”) AND TX (“Pseudomonas aeruginosa” OR “P. aeruginosa” OR pseudomonas) AND TX (biofilm* OR “bio-film” OR “bio film” OR “bacterial biofilm*” OR “microbial biofilm*” OR sessile OR “surface-attached” OR “surface attached”) AND TX (“Gram-negative” OR “Gram negative” OR “gram-negative bacteria” OR “gram negative bacteria” OR “GN bacteria” OR Enterobacteriaceae OR Proteobacteria OR “Escherichia coli” OR “Klebsiella pneumoniae” OR “Acinetobacter baumannii” OR “Salmonella spp” OR “Shigella spp”) = **17 ARTICLES**

WEB OF SCIENCE

TS = (pyocin OR pyocins OR "S-type pyocin" OR "R-type pyocin" OR "F-type pyocin" OR bacteriocin* OR "bacteriocin-like" OR colicin* OR microcin* OR "bactericidal protein*" OR "bactericidal peptide*") AND TS = ("Pseudomonas aeruginosa" OR "P. aeruginosa" OR pseudomonas) AND TS = (biofilm* OR "bio-film" OR "bio film" OR "bacterial biofilm*" OR "microbial biofilm*" OR sessile OR "surface-attached" OR "surface attached") AND TS = ("Gram-negative" OR "Gram negative" OR "gram-negative bacteria" OR "gram negative bacteria" OR "GN bacteria" OR Enterobacteriaceae OR Proteobacteria OR "Escherichia coli" OR "Klebsiella pneumoniae" OR "Acinetobacter baumannii" OR "Salmonella spp" OR "Shigella spp"). = **43 ARTICLES**
